# An NCR1-based chimeric receptor endows T-cells with multiple anti-tumor specificities

**DOI:** 10.18632/oncotarget.1919

**Published:** 2014-04-24

**Authors:** Yair Tal, Shlomo Yaakobi, Miryam Horovitz-Fried, Einav Safyon, Benyamin Rosental, Angel Porgador, Cyrille J. Cohen

**Affiliations:** ^1^ Laboratory of Tumor Immunology and Immunotherapy, The Goodman Faculty of Life Sciences, Bar-Ilan University, Ramat Gan 52900, Israel; ^2^ The Shraga Segal Department of Microbiology, Immunology and Genetics, and the National Institute for Biotechnology in the Negev, Faculty of Health Sciences, Ben-Gurion University of the Negev, Beer-Sheva 84105, Israel

**Keywords:** NCR1, Tumor Immunotherapy, T-cells, T-cell engineering

## Abstract

The Ral (Ras-like) GTP-binding proteins (RalA and RalB), as effectors of the proto-oncogene Natural killer (NK) cells are an important component of the anti-tumor response. Tumor recognition by NK cells was found to be partly triggered by molecules termed natural cytotoxic receptors (NCRs). Adoptive transfer of genetically-engineered tumor-reactive T-lymphocytes can mediate remarkable tumor regressions mostly in melanoma and leukemia patients. Yet, the application of such treatments to other cancers is needed and dependent on the isolation of receptors that could facilitate efficient recognition of these malignancies. Herein, we aimed at combining NK tumor recognition capability with the genetic modification of T-cells to provide the latter with a means to recognize several tumors in a non-MHC restricted way.

Consequently, we generated and evaluated several chimeric receptors based on the extracellular domain of NCR1 (NKp46) fused to multiple signaling moieties and assess their antitumor activity when retrovirally expressed in T-cells. Following co-culture with different tumors, primary human T-lymphocytes expressing a chimeric NCR1 molecule recognized target cells derived from lung, cervical carcinoma, leukemia and pancreatic cancer. In addition, this receptor mediated an upregulation of surface activation markers and significant antitumor cytotoxicity both i*n vitro* and *in vivo*. These results have meaningful implications for the immunotherapeutic treatment of cancer using gene-modified T-cells.

## INTRODUCTION

Tumor development and progression has often been reported to be associated with the lack of specific recognition of cancer cells by the immune system [1]. Natural killer (NK) cells are an important component of the anti-tumor response and their lytic capability depends on the integrated balance between activating and inhibitory signals [2;3]. Engagement of inhibitory receptors by MHC class I can prevent NK cell activation, thereby protecting the target cells from NK cell attack and thus, NK function is triggered in part by the recognition of “missing self” [4]. In addition, positive signals mediated by receptors are needed for full NK activation and tumor cell lysis [5]. Activating receptors include NKG2D, DNAM-1 and the natural cytotoxicity receptors (NCRs) family, the latter being composed of three molecules: NCR1 (NKp46), NCR-2 (NKp44) and NCR-3 (NKp30) [6]. NCR1 was the first NCR isolated and is a type I-transmembrane receptor that contains 2 Ig-domains and that associates with either CD3ζ or FcμRIγ in order to signal [7;8]. We and others showed that NCR1 is central to the recognition and lysis by NK cells of viral-infected and tumor cells such as carcinomas, neuroblastomas, and leukemias [9-16]. Though it has been demonstrated that heparan sulfate, which can be upregulated by cancer cells, can bind to NCR1 [17], the precise identification of the cellular ligands of NCR1 remains a challenge [6;18].

So far, the use of NK cells for immunotherapy-based cancer treatments has been limited due in part to difficulties in expanding and properly conditioning these lymphocytes [19], poor reactivity *in vivo* using autologous approaches [20] and immune rejection in allogeneic settings [21;22]. Conversely, the adoptive transfer of another type of tumor-reactive cells - T-lymphocytes - has been demonstrated to mediate the regression of large solid and hematological tumors in cancer patients [23;24]. In that regard, we and others have shown that it is possible to engineer lymphocytes to express T-cell receptors (TCRs) that confer novel anti-tumor activity directed against various types of cancer [25]. Still, the use of this therapeutic approach is limited to patients that express the appropriate MHC molecule to be recognized by the genetically introduced anti-tumor TCR. Provided the target antigen is expressed on the surface of the tumor cell, it is possible to circumvent this using chimeric receptors composed of a targeting moiety (usually an antibody fragment specific for a defined antigen) and a signaling portion (derived from CD3ζ or FcRIIIγ molecules) [23]. However, these strategies are often directed at a specific antigen whose expression may be restricted to certain types of cancer.

Thus in the present study, we combined the therapeutic potential of gene-modified T-cells with the recognition pattern of NCR1 in order to devise a targeting strategy directed towards multiple tumors in a non-MHC restricted way. We designed and optimized an NCR1-based chimeric receptor. The latter endowed primary human T-cells with anti-tumor activity against various malignancies by means of cytokine secretion, upregulation of activation markers, improved expansion and cytotoxicity *in vitro* and in a mouse model.

## RESULTS

### Construction and evaluation of NCR1-based chimeric receptors

**Figure 1:**
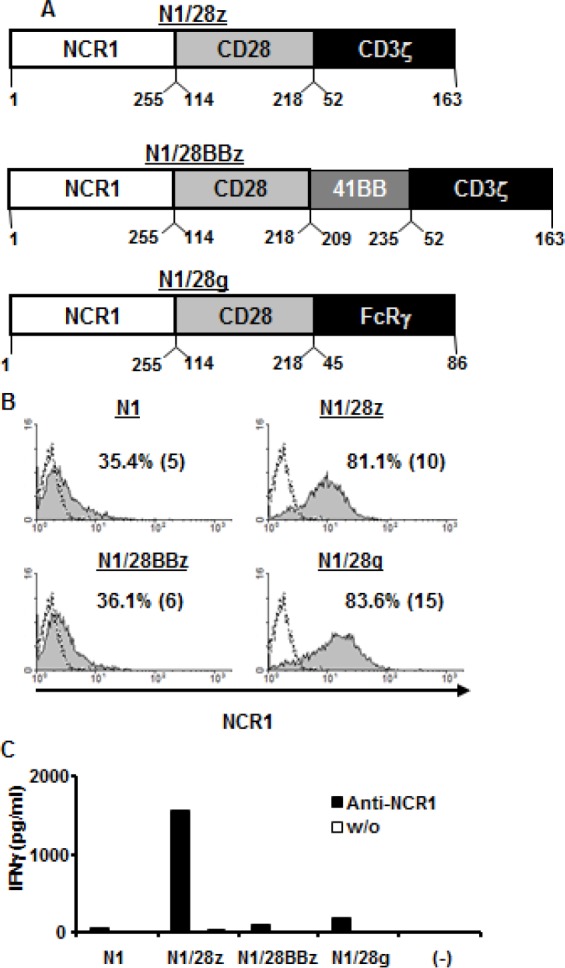
Design and expression of NCR1 chimeras (A) – Schematic representation of the different NCR-1-based chimeric receptors. The amino acid numbering (based on the original protein) is indicated below each segment. (B) Jurkat RT3-T3.5 cells were electroporated with 2 μg of mRNA encoding the wild-type NCR1 (N1) and its chimeric versions as indicated. NCR-1 expression was assessed by flow cytometry 24 h after electroporation. The dotted line represents the NCR1-staining of the mock-electroporated control. The percentage of positive cells and the MFI (in brackets) are shown. These results are representative of 4 independent experiments and the difference between the population transduced with NCR1 chimeric molecules and the mock-electroporated population was found statistically significant (p<0.05; calculated using a *Student's* paired t-test). (C) Human primary lymphocytes were electroporated with 2 μg of mRNA encoding the different receptors as indicated. 10^5^ cells were incubated in a 96-well plate in the presence of plate-bound anti-NCR1 (0.2μg/well) for 16 h. IFNγ secreted in the co-culture supernatant was measured by ELISA. These results are representative of three independent experiments, performed with two different donors.

We generated various NCR1-based chimeric receptors by cloning out the cDNA encoding NCR1 from human NK-cells and by fusing its extracellular domain to different co-stimulatory/activating domains (Figure 1A). These and the wild-type NCR1 receptor (N1) were cloned into the pGEM-4Z/64A vector and we produced mRNA encoding these receptors which were expressed by electroporation into Jurkat cells. Twenty four hours after the electroporation, the expression of the different receptors was assessed by flow cytometry. As seen in Figure 1B, we were able to detect surface expression of all the introduced receptors, with N1/28z and N1/28g exhibiting the highest levels with 81.1 % (MFI=10) and 83.6 (MFI=15) of positive cells respectively, compared to the mock-electroporated background.

We then tested the function of these receptors by electroporating mRNA encoding the latter into OKT-3-stimulated human primary lymphocytes. These cells were incubated with plate-bound anti-NCR1 and after 16 h, we harvested the supernatant and measured IFNγ concentrations by ELISA. Of all the receptors tested, we found that N1/28z mediated the highest secretion of IFNγcompared to the unstimulated control (1565 vs. 30 pg/ml). Interestingly, whereas we observed a high level of surface expression for N1/28g, the latter performed relatively poorly in functional assays, suggesting that surface expression might not always be predictive of the receptor function. We thus selected N1/28z as our lead chimeric receptor for subsequent evaluation.

### N1/28z mediates the recognition of tumors of different histologies

**Figure 2:**
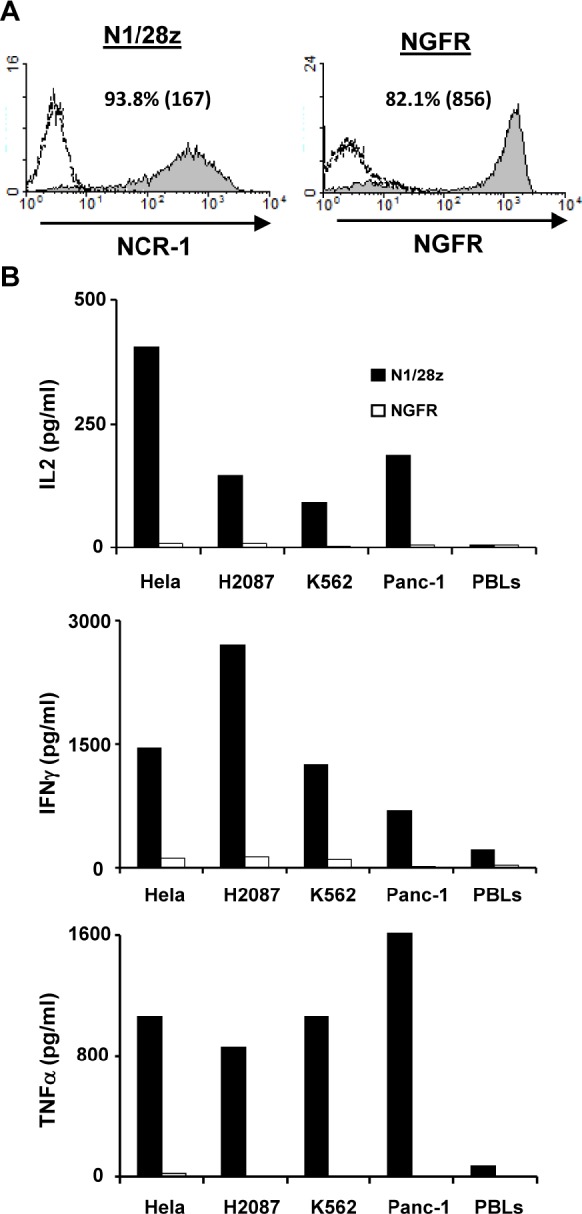
Figure 2: Anti-tumor function of the N1/28z chimera (A) Human primary lymphocytes were transduced with a retroviral vector encoding either N1/28z or NGFR (control). Transduced T cells were analyzed for NCR1 or NGFR expression 72 h after transduction by flow cytometry. The results are representative of 8 independent experiments performed with 6 different donors. (B) These cells were co-cultured with different tumor lines or normal PBLs as indicated. IL-2 (upper panel), IFNγ(middle panel) and TNFα (lower panel) secreted in the co-culture supernatant was measured by ELISA. These results are representative of four independent experiments, performed with three different donors and the difference between the N1/28z and NGFR populations was found statistically significant (p<0.05, calculated using a *Student's* paired t-test).

NCR1 has been shown to contribute to anti-tumor immunity [10;11;15;16]. To test whether our chimeric receptor N1/28z could mediate the recognition of tumors when expressed in primary human T-cells, we generated a retroviral construct based on the clinically-approved MSGV1 backbone and transduced primary human T-cells with retroviral supernatant encoding N1/28z or NGFR (control gene). These cells were stained with an anti-NCR1 antibody and analyzed by flow cytometry. As seen in Figure 2A, we achieved high levels of surface expression of the chimeric receptor 93.8% of positive cells with an MFI=167. These levels of expression were sustained for 30 days after transduction without selection and the growth and expansion of N1/28z-transduced cells was comparable to that of the NGFR-transduced population (data not shown).

Next, we assessed the antitumor function of the N1/28z receptor and set up a co-culture of N1/28z- or NGFR- (control) transduced cells with several tumor lines of different histology that express NCR1-ligand (Suppl. Fig. 1). We detected specific cytokine secretion by N1/28z-expressing cells compared to the NGFR-transduced control group (e.g. 1456 pg/ml vs. 95 pg/ml of IFNγ and 404 vs. 9 pg/ml of IL-2 respectively, in co-culture with HeLa cells; p<0.05 – Fig. 2B). No significant cytokine secretion was observed in co-culture with NGFR expressing cells or in the presence of normal PBLs. Thus, N1/28z can mediate the recognition of tumors of different histologies.

### Activation marker upregulation and cellular proliferation mediated by N1/28z

**Figure 3:**
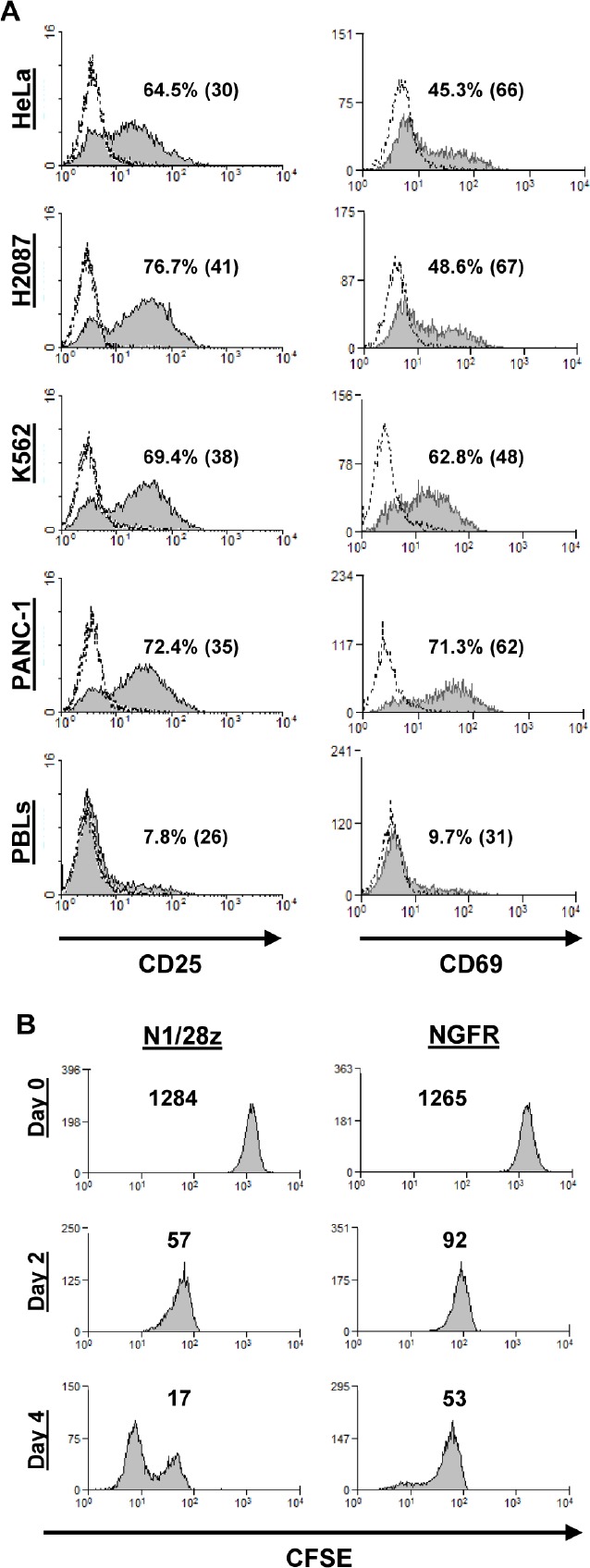
Activation marker upregulation and cell proliferation assay (A) Transduced PBLs with either N1/28z or NGFR cells were co-cultured with tumor lines as indicated and analyzed by flow cytometry for marker expression gated on the CD8^+^ population (CD25 – left panels; CD69 – right panels). The percentage of positive cells and the MFI (in brackets) are shown and the dotted line represents the marker staining of the NGFR control. These results are representative of at least four independent experiments with at least three donors and the difference between N1/28z and NGFR was found to be statistically significant (p<0.05, calculated using a *Student's* paired t-test). (B) *Proliferation assay.* CFSE-labeled activated transduced T-cells were stimulated with plate-bound anti-NCR1 (0.5 ug/well). Two and 4 days after stimulation, the cells were analyzed for CFSE dilution. The MFI is shown at the different time points. These results are representative of three independent experiments with two donors and the difference between the two groups was found statistically significant (p<0.05, calculated using a *Student's* paired t-test).

To test whether this NCR-based chimeric receptor could trigger the upregulation of T-cell activation markers, N1/28z- or NGFR- transduced T-cells were co-cultured overnight with different tumor lines and analyzed for surface expression of activation markers (CD25 and CD69). Compared to the NGFR-cell population, N1/28z-engineered cells demonstrated a statistically significant superior expression of these markers (Fig. 3A - e.g.: 69.4% (MFI=38) of CD25-positive cells and 62.8% (MFI=48) of CD69-positive cells in co-cultures with K562 tumor targets).

We also assessed the capacity of N1/28z to facilitate cellular proliferation upon triggering of the receptor. To do so, activated human T-cells, transduced to express N1/28z or NGFR, were labeled with CFSE and cultured in the presence of plate-bound anti-NCR1 for 4 days. These cells were analyzed for CFSE dilution on day 2 and 4 after the beginning of the stimulation. As seen in Fig. 3B, N1/28z-expressing lymphocytes proliferated more as demonstrated by the lower MFI of the analyzed population compared to NGFR-transduced cells (on day 2 – MFI=57 vs. 97 and on day 4 – MFI=17 vs. 53 respectively; p<0.05).

In conclusion, N1/28z can mediate the upregulation of activation markers and enhanced proliferation of transduced human T-lymphocytes.

### N1/28z chimeric receptor can mediate CD4^+^ T-cells activation

**Figure 4:**
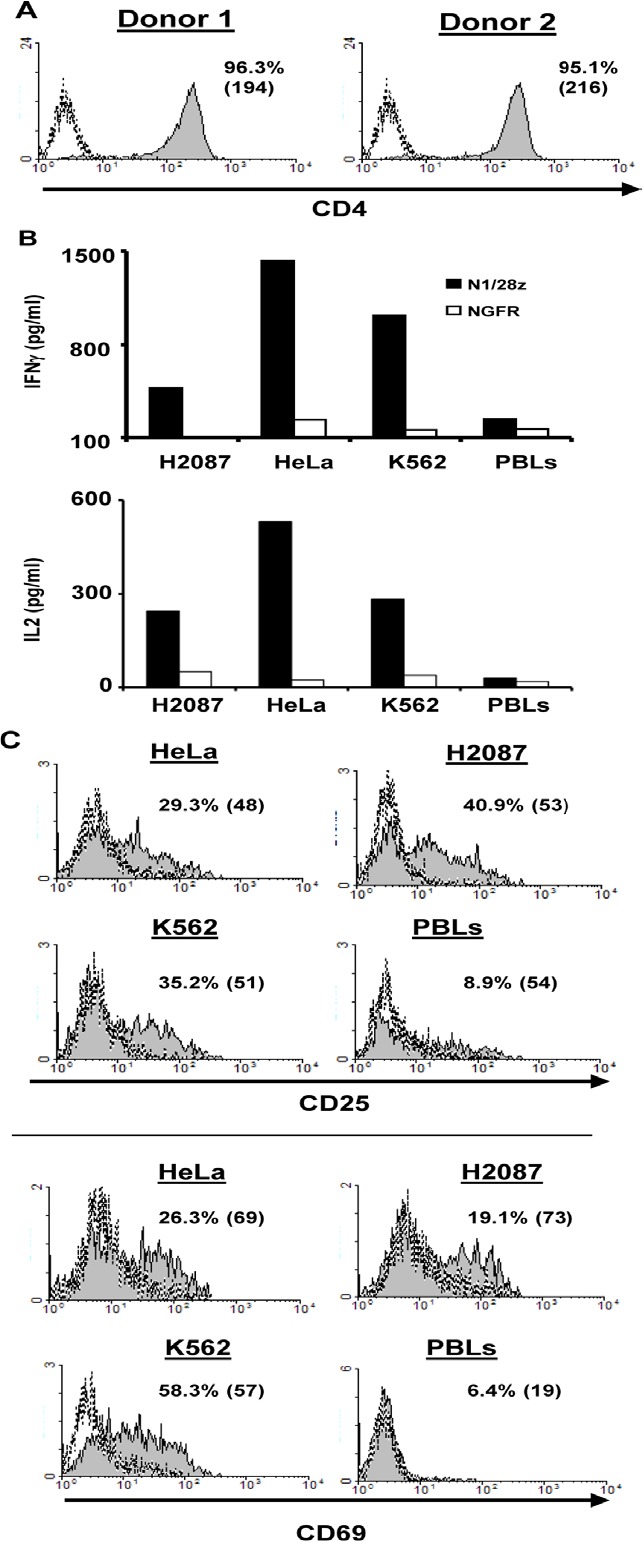
N1/28z function in CD4^+^ cells (A) Human CD4+ cells were purified using a magnetic beads approach. The purity of the separated population was analyzed by flow cytometry for two donors. The dotted line represents the fluorescence of the unstained population. The percentage of CD4^+^ positive cells and the MFI (in brackets) are shown. (B) N1/28z- or NGFR-transduced CD4^+^ cells were co-cultured with different tumor lines or not as indicated. IFNγ secreted in the co-culture supernatant was measured by ELISA. These results are representative of three independent experiments, performed with three different donors and the difference between the N1/28z and NGFR populations was found statistically significant (*: p<0.05, calculated using a *Student's* paired t-test). (C) CD4^+^-transduced T cells were analyzed by flow cytometry for marker expression (CD25 – upper panels; CD69 – lower panels) following overnight co-culture with tumor cells as indicated. The percentage of positive cells and the MFI (in brackets) are shown and the dotted line represents the marker staining of the NGFR control. These results are representative of three independent experiments with three donors and the difference between N1/28z and NGFR was found to be statistically significant (p<0.05, calculated using a *Student's* paired t-test).

CD4^+^ T-cell responses are essential to the full extent of the adaptive immune response. Unlike a classical T-cell receptor, the N1/28z chimeric receptor is not MHC-restricted, and thus we surmised it should function in CD4^+^ T cells. To test this, we purified human primary CD4^+^ cells (Fig. 4A) and transduced them with either N1/28z or NGFR. The cells were co-cultured with different tumor lines and analyzed for IFNγ and IL-2 secretion as well as activation marker upregulation. As expected, N1/28z mediated a significant cytokine secretion by transduced lymphocytes compared to the NGFR control (IFNγ - 1432 vs. 235 pg/ml and 1016 vs. 153 pg/ml in co-culture with HeLa and K562 respectively- Fig. 4B). Moreover, N1/28z caused an upregulation of the T-cell activation markers CD25 and CD69 as seen in Fig. 4C; e.g. 35.2% of CD25- and 58.3% of CD69-positive cells in co-culture with the K562 cell line. These data indicate that N1/28z can endow CD4+ T-lymphocytes with anti-tumor function.

### Cytotoxicity *in vitro* and *in vivo*

**Figure 5:**
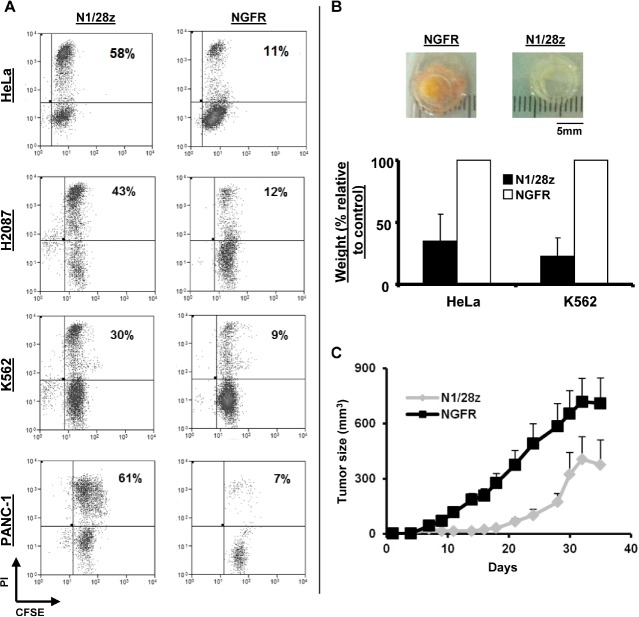
N1/28z mediates anti-tumor cytotoxic activity (A) N1/28z- or NGFR- transduced cells were co-cultured with the indicated CFSE-labeled tumor cells at a ratio of 1:1 (E:T). After 18 h, propidium iodide (PI) was added and the cells were analyzed by flow cytometry. The relative percentage of PI-positive cells (gated on the CFSE-labeled population - tumor cells) is shown. These results are representative of four independent experiments with two different donors and the difference between the N1/28z and NGFR populations was found statistically significant (p<0.05, calculated using a *Student's* paired t-test). (B) Tumors (derived from HeLa or K562 as indicated) that grew for five days on the CAM of chick embryo, were treated with adoptively transferred T-lymphocytes engineered to express either N1/28z or NGFR (control). At d=5-6 after treatment, the tumors were excised and weighed in a blinded manner. We show the percentage of the average tumor weight (+SEM) of the N1/28z-treated group relative to the NGFR-treated control group (100%). These results were obtained with three different donors and the difference between the N1/28z and the NGFR- (control) groups was found statistically significant (n=6; p=0.005, calculated using a *Student's* paired t-test). (C) *Winn assay.* Athymic nude mice were inoculated with HeLa cells and transduced lymphocytes (either N1/28z or NGFR –control as indicated) in the flank. Tumor growth was measured in a blinded fashion using a caliper and calculated using the following formula: (Dxd^2^ )xΠ/6, where D is the largest tumor diameter and d its perpendicular one. Results are shown for the different time points as mean+SEM of three separate experiments (n=3) that included 6 mice each, and the difference between the N1/28z and NGFR-treated groups was found statistically significant (p=3×10^−5^ ).

To ascertain the influence of the genetic modification of T-cells with N1/28z on T-cell cytotoxic potential, we first performed cell-mediated cytotoxicity assays; following the co-culture of transduced CD8^+^ T-cells with CFSE-labeled tumor cells, we observed a statistically significant anti-tumor cytotoxic activity mediated by N1/28z-transduced lymphocytes as exemplified by the PI-positive population (Fig. 5A - 58 % for N1/28z vs. 11 % for NGFR using the HeLa target cell-line; p=0.05).

The anti-tumor activity of N1/28z transduced T-cells was further confirmed in an *in ovo* cytotoxicity assay we recently developed [29;30], based on xenograft model using the chick embryo chorioallantoic membrane (CAM) model. Following treatment with N1/28z-transduced T-cells, we observed striking regressions of established tumors compared to control groups treated with NGFR-transduced T-cells (Fig. 5B). The average weight for tumors treated was reduced by approximately 65 % in the N1/28z treated tumors compared to the control treatment with NGFR cells (n=6; p=0.005). Finally, we performed a *Winn* assay to test the ability of N1/28z-transduced T-cells to mediate enhanced anti-tumor activity *in vivo*. Nude mice were inoculated with a mixture of HeLa cells and N1/28z- or NGFR-transduced cells (at E:T of 2:1) and we followed tumor development in the subsequent weeks. As seen in Fig. 5C, the N1/28z treated group displayed a statistically significant reduced tumor growth compared to the NGFR (control) group (p=3×10^−5^ ). In conclusion, the chimeric receptor N1/28z is able to mediate anti-tumor cytotoxicity both in *in vitro* and *in vivo* settings.

## DISCUSSION

Chimeric antigen receptor therapy is a promising approach for the treatment of advanced cancer [23;31]. As NCR1 has been shown to contribute to anti-tumor immunity [10;11;15;16], in the present work we have explored the use of NCR1 as a targeting moiety to redirect T-cells anti-tumor activity against a panel of different malignancies. While this receptor can associate with CD3ζ in order to signal [6] and recent studies suggest that certain T-cell subtypes can express NCR1 naturally (reviewed in Hudspeth *et al.* [8]), we did not observe a significant function mediated by the native NCR1 receptor when expressed in human primary T-cells (N1 receptor- Fig.1C). This lack of function might be partly explained by the need to compete with the T-cell receptor for CD3 molecules, the latter being considered a bottle-neck for TCR expression [32].

We designed several NCR1-based chimeric receptors and evaluated them in primary human T-cells. Whilst it has been argued that the inclusion of a 4-1BB signaling domain could improve T-cell function and prevent activation induced cell death [33;34], N1/28BBz showed a decreased stability (as suggested previously [31;35]) which apparently led to poor function (Fig.1C). Still, as the inclusion of co-stimulatory domains other than CD28 (e.g. OX40 [36] or CD27 [37]) may improve CAR-function, further optimization is warranted. In contrast, N1/28g which was expressed the most efficiently (Fig. 1B), failed to function (Fig. 1C) and this seems to be in agreement with previous observations [38;39]. Thus, the present results underscore the need to evaluate both the expression and the function of chimeric receptors empirically as previously advised [31;39].

After selecting an optimal construct, namely N1/28z, we expressed it in human primary T-cells and were able to target tumors of multiple origins. Such strategies aimed at targeting several malignancies using the same non-MHC restricted receptor [40-44] are attractive as they would provide an almost off-the-shelf “universal” reagent for the immunotherapeutic treatment of cancer. Unlike in NK cells in which NCR1 can be down-regulated in cancer patients [45;46] and its activity could be attenuated when exposed to tumor-expressed inhibitory ligands (such as MHC), T-cell expressing N1/28z could circumvent such an inhibition. On the other hand, since the nature of the cellular ligands of NCR1 is still a matter of intensive research [6], it will be important to include a suicide gene in the construct for potential clinical applications. As such, our *in vivo* assay (Fig. 5B) provides some preliminary insights into the therapeutic potential of the present approach.

As seen in Fig.4, N1/28z mediated also anti-tumor activity when expressed in CD4^+^ human primary T-cells. The possibility to recruit CD4^+^ T cells using an anti-tumor receptor is appealing as they may support CTL anti-tumor response [47]. However in clinical settings, one might need to utilize only selected helper T-cell populations such as Th1 and not Tregs or Th17 which have been shown respectively to hamper anti-tumor immunity or whose role in this context is unclear at the present.

It has been suggested that certain T-cell subtypes express NCRs as part of an original strategy to quickly respond to pathogens in a non-antigen specific manner [8]. In addition, we and others have shown that NCR1 may be central to the recognition and eradication of certain viral and bacterial infections [9;48-50]. Thus, we propose that our approach could be extended to the treatment of such infections using N1/28z redirected T-cells.

In conclusion, we have designed and optimized an NCR1-based chimeric receptor that enables the targeting of malignancies of diverse histology and we are confident that the development of such non-MHC restricted tumor-targeting strategies will be beneficial to adoptive transfer treatments based on engineered T- lymphocytes.

## MATERIAL & METHODS

### PBMCs and cell lines

All the PBMCs used in this study were from normal donors obtained from the Israeli Blood Bank (Sheba Medical Center, Tel-Hashomer, Israel). K562 (ATCC CCL-243) is an erythroleukemia cell line, H2087 (ATCC/CRL-5922) is a non-small cell lung cancer cell line, HeLa (ATCC/CCL-2) is a cervix adenocarcinoma cell line and PANC-1 (ATCC/CRL1459) is a pancreatic cancer cell line. Viral packaging cell line 293GP, which stably expresses GAG and POL proteins has been previously described [26]. Adherent cells were cultured in DMEM (Invitrogen, Carlsbad, CA), supplemented with 10% heat-inactivated Fetal Bovine Serum (Biological Industries, Beth Haemek, Israel) and were maintained in a 37°C and 5% CO_2_ incubator. Lymphocytes were cultured in BioTarget medium (Biological Industries, Beth Haemek, Israel) supplemented with 10% heat-inactivated FBS and 300 IU/ml IL-2 (Peprotech, Israel) and maintained at 37°C and 5% CO_2_.

### NCR1 chimeras and retroviral constructs

The cDNA encoding the human NCR1 was amplified from reverse-transcribed mRNA isolated from human NK cells. The different chimeras were created by overlapping PCR and their amino acid composition as indicated in Fig. 1A. These were cloned into the pGEM-4Z/64A vector which enables the *in vitro* generation of mRNA encoding the constructs as described previously [26;27]. The selected chimeric receptor, N1/28z as well as a truncated version of the long-nerve growth factor receptor (NGFR) were cloned into the well-characterized retroviral vector backbone pMSGV1 [28], which is a derivative of the MSCV-based splice-gag vector (pMSGV), and which uses a murine stem cell virus (MSCV) long terminal repeat.

### Electroporation of PBLs

This technique has been extensively described in our previous reports [26;27]. Briefly, *in vitro*-transcribed mRNA for NCR-1 chimeric receptor was electroporated into OKT3-stimulated PBLs at 400V/500μs using an ElectroSquare Porator ECM 830 (BTX, San Diego, CA). The amount of *in vitro*-transcribed mRNA was 2 μg per 1×10^6^ PBMCs.

### Transduction of PBLs

For virus production, transfection of 2×10^6^ 293GP cells with 9μg DNA of MSGV1-based retroviral construct and 4.5μg envelop plasmid (VSV-G) was performed using JetPrime transfection reagent (Polyplus, France). Retroviral supernatant was collected 36 h after the DNA transfection. Freshly isolated human PBLs were stimulated for 48 h in the presence of 50ng/ml OKT3 (eBioscience, San Diego, CA) before transduction. Following stimulation, lymphocytes were transduced with retroviral vectors by transfer to non-treated tissue culture dishes (Nunc, Rochester, NY) that had been pre-coated with RetroNectin (Takara, Japan) and retroviral vectors as previously described [26].

### FACS analysis and Antibodies

Fluorophore-labeled anti-human CD8, CD25, CD69 and NGFR were purchased from BioLegend (San Diego, CA). Anti-NCR1 antibody was purchased from R&D Systems (Minneapolis, MN). Immunofluorescence, analyzed as the relative log fluorescence of live cells, was measured using a CyAn-ADP flow cytometer (Beckman Coulter, Brea, CA). Approximately 1×10^4^ to 1×10^5^ cells were analyzed. Cells were stained in a FACS buffer made of PBS, 0.5% BSA, and 0.02% sodium azide.

### Cytokine release assays

Lymphocyte cultures were tested for reactivity in cytokine release assays using commercially available ELISA kits for IL-2, IFNγ and TNFα(R&D Systems, Minneapolis, MN). For these assays, 1×10^5^ responder cells (T-cells) and 1×10^5^ stimulator cells (tumor cells) were incubated in a 0.2-ml culture volume in individual wells of 96-well plates. Stimulator cells and responder cells were co-cultured for 18 h. Cytokine secretion was measured in culture supernatants diluted to be in the linear range of the assay. As a control for T cell activity, we incubated the different T-cell cultures with PMA/Ionomycin at a concentration of 50ng/ml and 1μM respectively.

### Cell proliferation assay

1×10^5^ T-cells were labeled with 1 μM CFSE (eBioscience, San Diego, CA) for 6 min and then cultured in 96-well plate in the presence of 0.5μg/well plate-bound anti-NCR1 antibody (R&D systems, Minneapolis, MN). On day 2 and 4 after stimulation, cell fluorescence was analyzed by flow cytometry.

### Cell separation

T-cell populations were separated using a magnetic beads-based approach for negative selection (EasySep ^TM^ - StemCell Technologies Inc., Canada).

### Cell Mediated Cytotoxicity Assay

Target cells were labeled with 2 μM CFSE (eBioscience, San Diego, CA) for 6 min and then co-cultured with transduced lymphocytes at 37ºC for 18 h, at E:T ratio of 1:1. After the co-culture, propidium iodide (PI) 1μM (Sigma-Aldrich, Jerusalem, Israel) was added for assigning the ratio of cell death. Samples were analyzed by flow cytometry.

### Winn Assay

Six weeks-old athymic nude-Foxn1nu female mice (Harlan, Jerusalem, Israel) were inoculated in the flank with a mixture of 10^6^ HeLa cells and 2×10^6^ transduced lymphocytes resuspended in 40 μl Biotarget medium. Tumor size was measured every 3 days using a caliper in a blinded fashion. All the procedures were performed according to the guidelines of the university committee for animal welfare.

## SUPPLEMENTARY MATERIAL FIGURE


