# Supplementary material for: An NCR1-based chimeric receptor endows T-cells with multiple anti-tumor specificities
Source: Oncotarget. 2014 Apr 24;5(21):10949–58. doi: 10.18632/oncotarget.1919 (PMC4279421; doi:10.18632/oncotarget.1919)
Supplement: Supplementary file 1 [file oncotarget-05-10949-s001.pdf]

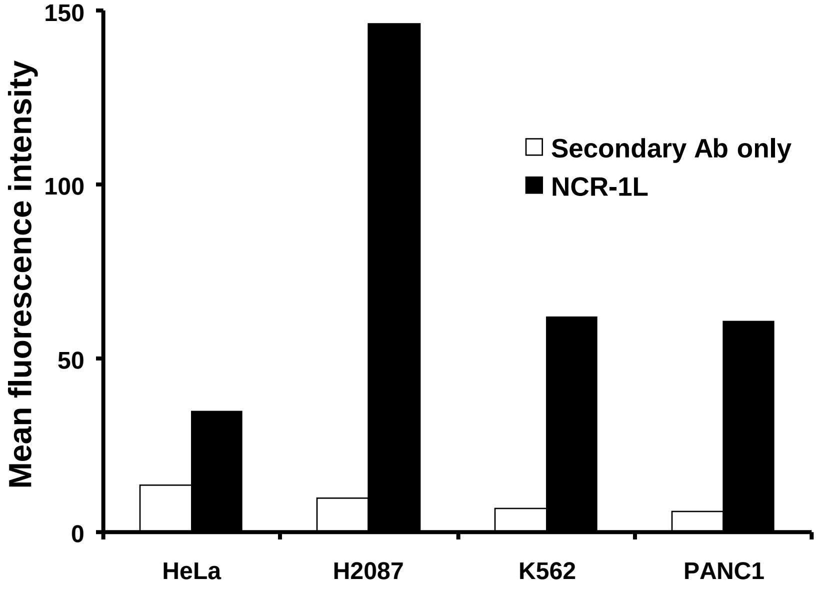

Supplementary Figure 1: *Expression of NCR-1 ligands by tumor cells.* 0.5 x 10^6 tumor cells (as indicated) were incubated with an Fc-NCR1 fusion protein, followed by a fluorescently-labeled anti-Fc secondary antibody (black bars) or only with the secondary antibody (white bars - control). The samples were analyzed by flow cytometry.
